# Factors associated with perceived quality of life in artisanal fishermen: a cross-sectional study

**DOI:** 10.1186/s13104-019-4525-4

**Published:** 2019-08-02

**Authors:** Bhárbara Karolline Rodrigues Silva, Francisco Winter dos Santos Figueiredo, Erika da Silva Maciel, Fernando Rodrigues Peixoto Quaresma, Fernando Adami

**Affiliations:** 1 Epidemiology and Data Analysis Laboratory, Faculdade de Medicina do ABC (FMABC), Santo André, SP Brazil; 2 Universidade Federal do Tocantins (UFT)–Campus de Miracema, Miracema do Tocantins, Tocantins Brazil; 3 Universidade Federal do Tocantins (UFT)–Campus de Palmas, Palmas, Tocantins Brazil

**Keywords:** Artisanal fishermen, Vulnerable populations, Quality of life, Chronobiology, Stress

## Abstract

**Objective:**

To analyze a combination of socioeconomic and demographic characteristics, chronotype, stress perception and level of physical activity with the perception of quality of life in artisanal fishermen.

**Results:**

Several variables were associated with lower scores of quality of life domains: workload (P = 0.047), age (P = 0.01), economic class D–E (P = 0.04) and perceived stress scores (P = 0.01) for scores physical domain; workload (P = 0.03) and perceived stress (P < 0.001) for scores psychological domain; Perceived stress (P < 0.001) and age (P = 0.01) for social domain scores; economic class D–E (P = 0.03) and perceived stress (P = 0.01) for environment domain scores; perceived stress (P = 0.01) and age (P = 0.01) for general quality of life scores and; female sex (P = 0.04) and age (P = 0.02) for the score of satisfaction with quality of life.

## Introduction

Worldwide there were more than 43.5 million fishermen in 2009 [1]. In Brazil, there were more than 970 thousand fishermen in 2014, of which approximately 957 thousand were artisanal fishermen [2], characterized by fishing autonomously, preserving socio-cultural traditions, with family regime, as a source of income, through their own production or in partnership, in small vessels with little autonomy [3, 4]. This type of activity has great socioeconomic importance for several countries such as China, India, Indonesia, Japan and Chile [5, 6], as it represents employment and food security [3, 6–9].

They commonly work in precarious labor environments, with increased workloads and exposure to factors that compromise health, such as stress, insufficient physical activity, and shifts when biological predisposition (chronotype) is not respected. Chronotype can influence sleep quality, labor productivity, social performance and disease development, such as cardiovascular and metabolic responses that are detrimental [10–17].

Socioeconomic and demographic vulnerabilities due to individual and/or collective issues, as unavailability to access resources, health services, potable water, sanitary sewage are factors which can increase susceptibility to diseases and impact on quality of life (QOL) [14, 16–19].

However, few studies analyze the perception of the quality of life and associated factors in vulnerable populations. Public policies to improve the health and right of the minority communities’ need of the scientific evidences to improve it, but scientific evidences about this are gaps in the actual literature [20, 21]. This situation makes artisanal fishermen less aware of their QOL when compared to the general population [14, 16].

In this sense, what factors can interfere in the diminished of the perception of the QOL of artisanal fishermen? The objective of this study was to analyze the association between socioeconomic and demographic characteristics, chronotype, stress perception and level of physical activity with the QOL perception in artisanal fishermen.

## Main text

### Methods

Cross-sectional study reported according to Strengthening the Reporting of Observational Studies in Epidemiology (STROBE) [22]. Performed in the state of Tocantins, north of Brazil, between 2016 and 2017.

In the Tocantins state (north of Brasil), there are 36 fishermen colonies registered in the Institute of Rural Development of the State of Tocantins (RURALTINS). Among these, the colonies “Itaobi”, “Z-10”, “Parque Sucupira “ and “Colônia de Pescadores de Miracema do Tocantins (COPEMITO)” were selected for this study. The selected colonies are located around the watershed of the Tocantins River, second largest river in Brazil with 172,828 km^2^ and with great significance for the state economy for allowing besides fishing, aquaculture practice and tourism activities [23, 24].

The project was approved by the Committee of Ethics in Research with Human Subjects (no 50419215.5.0000.5516). Access to the colonies was obtained by means of a partnership with the Brazilian Company of Agricultural Research-Fishermen and Aquaculture (EMBRAPA). The collections were performed by previously trained staff, through face-to-face interviews.

Were evaluated for eligibility all artisanal fishermen registered in the selected colonies (n = 175), with 18 years or more. Those who refused to sign an informed consent form (n = 20) or withdraw consent during the data collection phase (n = 10) were not included. In total, 145 artisanal fishermen composed the sample, and 01 were excluded due to inadequate data filling.

The socioeconomic and demographic characteristics were obtained by data collection form prepared by the authors. The Economic Classification was evaluated through the Questionnaire of the Brazilian Association of Research Companies (ABEP). The Economic Classification was evaluated through the Questionnaire of the Brazilian Association of Research Companies (ABEP). Composed of 15 questions, which assess the quantity of automobiles, bathrooms, appliances, electronics, employees, educational level, origin of water used in the home and whether the stretch of the street that resides is paved. The socioeconomic level was classified according to the Brazilian Economic Classification Criteria (Classes A, B, C, and D–E) that represents the socioeconomic strata of the population (e.g. class A has better education, income, life conditions while Class D–E has poorest education, income, as well other socioeconomic indexes assessed by the questions) [25].

Perceived stress, measured by the EPS-10 Stress Perceptual Scale considered as a global stress measure, validated for the Brazilian population [26, 27]. This instrument assesses the perception of stress in certain situations within the last 30 days, and is related to a series of self reports and behavioral criteria. The instrument is composed of 10 items evaluated on a Likert scale, and the final result corresponds to a score from 0 to 40. The higher the final score the greater the perceived stress [27].

To evaluate the chronotype of the participants, the Morning and Evening Individual Identification Instrument [26, 28] was used, which classifies them in the Morning, Moderately Morning, Intermediate, Evening and moderately evening, according to the individual’s preferred daily habits [29, 30]. Individuals classified as morning have a greater predisposition to sleep and wake up early, with better work performance and alert level in the morning; those classified as intermediaries have better flexibility, can adapt to schedules according to needs, and evening individuals have a preference for sleeping and waking up late, with better performance in the afternoon and early evening [26, 31, 32].

Physical Activity Level was evaluated by the International Physical Activity Questionnaire (IPAQ) version 8, short form and normal week. The participants were classified as having low, moderate or high level of physical activity [33, 34].

The QOL perception was evaluated through the WHOQOL-bref instrument (World Health Organization Quality of Life), developed and validated by the World Health Organization (WHO), composed of 26 facets. The facets are divided into 4 domains of quality of life, which are physical, psychological, social relations and environment. Self-perception is assessed through the mean scores of the domains and the higher the score, the better the QOL perception [35–37].

The QOL perception and perceived stress are self-reported and subjective measures that can be influenced by psychological phenomena, discernment of the values they are inserted and by personal judgment itself [37, 38]. This subjectivity and possibility of influence by external factors can generate information bias. Measures such as the training of interviewers and the use of validated instruments for the Brazilian population were carried out to reduce this bias.

Due to the lack of studies on the QOL of artisanal fishermen, the QOL parameter of a study with a resident population in one of the study cities (Palmas-TO) was used to estimate the sample size calculation, having as parameter the greatest variability found of the indicators analyzed.

The standard deviation of the general QOL score of 3.8 was used based on a population of 369 people [39], power of 80%, 95% confidence level, 5% sampling error, and a total of 323 fishermen registered in the studied communities, a sample of 132 artisanal fishermen would be necessary. The sample was increased by 10% for eventual losses related to data collection, and the final sample size was 145 artisanal fishermen.

Descriptive statistic was performed. Additionally, Shapiro–Wilk test was used to analyze the adherence to the normal distribution. When they did not present a normal distribution, logarithmic transformation was performed so that all variables presented normal distribution (Shapiro–Wilk, p ≥ 0.05) and parametric tests were used. Linear regression models were created. The stepwise backward strategy was used, with variables entry into the model p = 0.20 and removal p = 0.05. Missing were not considered in the analyzes. The response rate of the variables was less than 20%. The level of significance was 5% and Stata ^®^ (StataCorp, LC) 11.0 was used.

### Results

Of 144 artisanal fishermen studied, the majority were men (70.83%, n = 102), worked for more than 12 h a day (67.36%, n = 97), worked as artisanal fishers for up to 10 years (53.47%; n = 77) and had only one employment relationship (60.42%; n = 87) (Table 1).

**Table 1:** Socioeconomic demographic characteristics, chronotype, level of physical activity and perceived stress

Variables	n	%
Sex		
Female	42	29.17
Male	102	70.83
Daily workload		
Up to 12 h	47	32.64
> 12 h	97	67.36
Time of profession		
Up to 10 years	77	53.47
> 10 years	67	46.53
Number of employment relationship		
1	87	60.42
> 1	57	39.58
Economic class		
A	4	4.00
B	23	23.00
C	27	27.00
D–E	46	46.00
Smoking		
Not	86	60.56
Yes	56	39.44
Chronotype		
Moderately afternoon	4	2.82
Intermediate	135	95.07
Moderately morning	3	2.11
Physical activity level		
Low	39	27.08
Moderate	49	34.02
High	56	38.90

	Mean	Standard deviation
Age	50.10	8.99
Monthly income (USD)	427.89	386.34
Perceived stress score	29.07	5.11

USD: dollar

Fishermen with economic class D or E (46.0%; n = 46), classified as intermediate chronotype (95.07%; n = 135), with high level of physical activity (38.62%; n = 56); and non-smokers (60.56%, n = 86) characterized most of the sample. On mean (standard deviation, sd), fishermen presented 50 (8.99) years, monthly income of USD $ 427.89 (386.34) and perceived stress of 29.07 (5.11) (Table 1).

The mean (sd) of the general QOL perception of artisanal fishermen were 63.98 (18.43) and the QOL satisfaction of 60.41 (26.52), on the scale transformed to 0 to 100. In relation to the scores of the QOL domains, on mean (sd) artisanal fishermen presented a score of 74.35 (15.29) for social relations, 71.76 (13.39) for the psychological domain, 66, 22 (15.43) for the physical domain and 56.31 (12.22) for the domain environment (Table 2).

**Table 2:** Scores of the domains of the WHOQOL-bref

Domains	Mean	SD	Min	Max
Social relationships	74.35	15.29	16.66	100
Psychological	71.76	13.39	25.00	100
Physicist	66.22	15.43	17.85	100
Environment	56.31	12.22	12.50	78.1
General quality of life	63.98	18.43	0.00	100
Overall satisfaction with quality of life	60.41	26.52	0.00	100

*SD* standard deviation, *Min* minimum value, *Max* maximum value

The factors associated with each domain of the QOL perception with the general QOL score and with the satisfaction with the fishermen QOL were presented in Table 3.

**Table 3:** Association between socioeconomic demographic characteristics and perception of quality of life

Domains	Linear regression	
	β (95% CI)	p*
Physical domain		
Sex	− 0.81 (− 1.83; 0.21)	0.12
Workload	− 1.00 (− 1.99; − 0.01)	0.047
Time of profession	0.65 (− 0.24; 1.55)	0.15
Age	− 0.91 (− 0.14; − 0.04)	0.01
Economic classification		
A	Ref.	Ref.
B	− 1.39 (− 3.74; 0.95)	0.24
C	− 1.97 (− 4.35; 0.39)	0.10
D–E	− 2.40 (− 4.69; − 0.10)	0.04
Physical activity level		
Low	Ref.	Ref.
Moderate	0.84 (− 0.26; 1.96)	0.13
High	0.94 (− 0.14; 2.03)	0.08
Perceived stress	− 0.14 (− 0.24; − 0.05)	0.01
Psychological domain		
Sex	− 16.32 (− 31.90; 4.68)	0.48
Workload	− 28.29 (− 39.21; − 8.01)	0.03
Economic classification		
A	Ref.	Ref.
B	− 17.11 (− 45.26; 38.24)	0.74
C	− 12.31 (− 44.04; 40.45)	0.86
D–E	− 37.07 (− 55.67; 18.71)	0.11
Age	− 6.06 (− 8.62; 0.77)	0.05
Physical activity level		
Low	Ref.	Ref.
Moderate	16.55 (− 23.66; 33.29)	0.52
High	23.39 (− 16.55; 37.0)	0.19
Perceived stress	− 11.83 (− 14.50; − 8.36)	< 0.001
Social domain		
Perceived Stress	− 13.04 (− 16.00; − 9.18)	< 0.001
Age	− 7.68 (− 10.25; − 3.60)	0.01
Time of profession	26.16 (− 12.40; 39.02)	0.10
Income	0.25 (− 0.58; 0.46)	0.65
Economic class		
A	Ref.	Ref.
B	28.90 (− 39.32; 56.71)	0.49
C	19.02 (− 45.67; 53.00)	0.77
D–E	− 14.10 (− 51.10; 47.05)	0.87
Environment domain		
Economic class		
A	Ref.	Ref.
B	− 30.08 (− 46.91; 19.79)	0.17
C	− 32.89 (− 48.91; 15.11)	0.10
D–E	− 37.64 (− 51.73; − 12.56)	0.03
Physical activity level		
Low	Ref.	Ref.
Moderate	9.15 (− 23.35; 26.69)	0.79
High	17.11 (− 17.51; 29.87)	0.33
Perceived stress	− 8.87 (− 11.42; − 5.18)	0.01
Number of employment links	15.17 (− 17.05; 27.41)	0.38
General quality of life		
Income**	3.68 (1.29; 6.08)	0.01
Perceived stress	− 0.82 (− 1.54; − 0.09)	0.03
Time of profession	5.49 (− 1.65; 12.63)	0.13
Age	− 0.51 (− 0.90; − 0.01)	0.01
Economic classification		
A	Ref.	Ref.
B	10.40 (− 10.02; 30.82)	0.32
C	8.49 (− 12.50; 29.47)	0.42
D–E	− 2.28 (− 23.00; 18.41)	0.83
Satisfaction with quality of life		
Sex	− 12.55 (− 24.70; − 0.39)	0.04
Age	− 0.69 (− 1.29; − 0.09)	0.02
Time of profession	7.92 (− 3.02; 18.86)	0.15
Economic class		
A	Ref.	Ref.
B	− 9.08 (− 37.85; 19.70)	0.53
C	− 11.28 (− 39.95; 17.38)	0.44
D	− 15.55 (− 43.50; 12.40)	0.27

Ref.: Reference variable

* Linear regression

** Variation of USD 100.00

Sex was associated only with the satisfaction of fishermen with the QOL perception. Based on the male sex, female artisanal fishers had a lower QOL satisfaction score (β = − 12.55, 95% CI − 24.70 to − 0.39, p = 0.04). Age was associated with QOL scores in the physical domains (β = − 0.91, 95% CI − 0.14 to − 0.04, p = 0.01) and social relationships (β = − 7.68, 95% CI − 10.25 to − 3.60, p = 0.01), to the general QOL (β = − 0.51, 95% CI − 0.90 to − 0.01, p = 0.01) and satisfaction with QOL (β = − 0.69, 95% CI − 1.29 to − 0.09, p = 0.02). There were only statistically significant differences among fishermen belonging to the D–E classes when compared to class A for the physical domains (β = − 2.40, 95% CI − 4.69 to − 0.10, p = 0.04) and environment (β = − 37.64, 95% CI − 51.73 to − 12.56, p = 0.03).

The stress perceived was one of the main factors associated to the decrease of QOL in all domains: social relations domains (β = − 13.04, 95% CI − 16.00 to − 9.18, p < 0.001); psychological (β = − 11.83, 95% CI − 14, (β = − 8.87, 95% CI − 11.42 to − 5.18, p = 0.01); physical (β = − 0.14, 95% CI − 0.24 to − 0.05, p = 0.01) and; in general QOL (β = − 0.82, 95% CI − 1.54 to − 0.09, p = 0.03) (Table 3).

Based on the workload of less than 12 h a day, fishermen working 12 or more hours per day have lower QOL perception in the physical domain (β = − 1.00, 95% CI − 1.99 to − 0.0, p = 0.047) and in the psychological domain (β = − 28.29, 95% CI − 39.21 to − 8.01, p = 0.03) (Table 3).

### Discussion

The factors associated with the QOL perception of artisanal fishermen were female sex, age, economic classification, workload and perception of stress. The main ones’ alert to the impact of perceived stress, age and income on the QOL perception.

The findings of this study seem to be the first related to the perception of quality of life Brazilian artisanal fishermen, as well as the factors that may influence this perception. The main ones alert to the impact of perceived stress, age and income on the perception of quality of life. In this sense, the lower income, greater perceived stress and greater age, the lower the quality of life perception scores of artisanal fishermen.

#### Physical domain

Age and economic status were factors associated with lower perception of quality of life in the physical domain. The physical domain consists of 12 facets, such as pain and discomfort, energy and fatigue, sleep and rest, and work capacity [40].

The lower socioeconomic status is related to the need for more hours of work and, consequently, greater stress. These factors together with old age are associated with lower scores in the physical domain [40]. Factors together with old age are associated with lower scores in the physical domain [40], therefore older artisanal fishermen had lower scores of perceived QOL [41].

With increasing age, physiological functions and aptitudes and mental abilities trend to decrease. Consequently, the individual’s energy decreases [42], impacting on lower energy and higher fatigue scores. Artisanal fishermen with insufficient monthly income must increase productivity for higher income, spend more time fishing, results in increased fatigue and reduced work capacity [15].

Thus, the more time in of profession in fishing, the more likely to arise musculoskeletal disorders to be a profession that involves ergonomic risks related to the management of excess weight, increased exposure to ultraviolet rays that can cause diseases, such as cancer, burns, among others [41, 43]. With these factors there is also increased the dependence on medication or treatments, which have repercussions on the facet pain, discomfort, ability to work.

#### Psychological domain

The perceived stress and the workload over 12 h were the factors associated with lower scores for psychological domain. The increased workload causes mental fatigue and becomes the main source of stress [44, 45]. The consequences of stress in relation to work may be discouragement, anxiety, interpersonal difficulties, lack of interactivity in relation to work activities, fear of the dangers found in the water, among other health problems [45, 46], situations that may also impact on the psychological domain.

#### Social domain

Age and perceived stress were the factors associated with lower QOL perception in the social domain. This domain consists of facets such as personal relationships, sexual activity, social support, these can be influenced by stress. The lack of support for the profession and policies to encourage small-scale fishing are a reality experienced by these fishermen, especially among older fishermen. In this scenario, fishing activity is weakened, giving rise increase in labor stress and consequent reduction of QOL [9, 47].

#### Environmental domain

Perceived stress and the socioeconomic level were the factors that reduced the QOL perception in the environmental domain. The artisanal fishermen has as main source of income the fishing and when this is not productive there is decrease of per capita income [48]. Other factors that contribute to fishermen productivity are infrastructure, irritating noises, declining fish species, lack of institutional support and policies aimed at fishing, may lead to deactivation of the activity, and become development of stress [43, 47, 49–51].

The fact that the fishermen stays most of the time in the river for hours or days away from home, in an environment that is precarious hygiene and health care, where there is no physical security and protection, short of the natural adversities of the environment and the fact that this profession is among the most dangerous [17, 43]. This may negatively interfere with the perception of stress [52], as well as the reduction in the related to facet physical security and protection.

#### Generalisability

The quantity of artisanal fishermen in the world and the way they preserve their culture and habits, makes this population unique and significant. The state of Tocantins, where the study was conducted, is the newest Brazilian state that is in constant development. Little is known about the life habits, quality of life, chronotype and perceived stress of artisanal fishermen, and the results presented in this study are important for a better understanding and to obtain estimates of these indicators in this vulnerable population.

The main factors associated with a lower perception of quality of life were related to aspects that can be improved with low cost and easy to implement public policies, such as improving access to sanitary sewage, expanding coverage of health strategy family and the maintenance of income redistribution policies. In general, providing health promotion policies to artisanal fishers aimed at improving income, the perception of quality of life over the age and the reduction of stress can produce positive effects on well-being, consequently in the quality of life score general. Thus, existing public policies and new public policies related to the health of artisanal fishers must consider the specific needs of each region, mainly due to the socioeconomic disparities and inequalities in Brazil.

## Limitations

Several limitations can be related to the study design, sample size and outcome measurement. The study design is the major limitation of this study, since they may influence the external validity of the data and make it impossible to establish a cause and effect. The QOL perception and perceived stress are self-reported and subjective measures, and can limit the extrapolation these data for different fishermen colonies. Additionally, founds of this study should be viewed with caution because of sample size. However, they were obtained from a sample size of the four fishermen colonies, and the full research will include 36 colonies.

## Data Availability

Not applicable.

## Abbreviations

ABEP: Brazilian Association of Research Companies
COPEMITO: Colônia de Pescadores de Miracema do Tocantins
EMBRAPA: Brazilian Company of Agricultural Research-Fishermen and Aquaculture
IPAQ: International Physical Activity Questionnaire
QOL: quality of life
RURALTINS: Rural Development of the State of Tocantins
STROBE: Strengthening the Reporting of Observational Studies in Epidemiology
USD: dollar
